# New strategic insights into managing fungal biofilms

**DOI:** 10.3389/fmicb.2015.01077

**Published:** 2015-10-06

**Authors:** Elisa Borghi, Giulia Morace, Francesca Borgo, Ranjith Rajendran, Leighann Sherry, Christopher Nile, Gordon Ramage

**Affiliations:** ^1^Laboratory of Microbiology, Department of Health Sciences, Università degli Studi di Milano, Milan, Italy; ^2^Infection and Immunity Research Group, Glasgow Dental School, School of Medicine, University of Glasgow, Glasgow, UK

**Keywords:** biofilm-related infections, antifungal resistance, myriocin, fulvic acid, acetylcholine

## Abstract

Fungal infections have dramatically increased in the last decades in parallel with an increase of populations with impaired immunity, resulting from medical conditions such as cancer, transplantation, or other chronic diseases. Such opportunistic infections result from a complex relationship between fungi and host, and can range from self-limiting to chronic or life-threatening infections. Modern medicine, characterized by a wide use of biomedical devices, offers new niches for fungi to colonize and form biofilm communities. The capability of fungi to form biofilms is well documented and associated with increased drug tolerance and resistance. In addition, biofilm formation facilitates persistence in the host promoting a persistent inflammatory condition. With a limited availability of antifungals within our arsenal, new therapeutic approaches able to address both host and pathogenic factors that promote fungal disease progression, i.e., chronic inflammation and biofilm formation, could represent an advantage in the clinical setting. In this paper we discuss the antifungal properties of myriocin, fulvic acid, and acetylcholine in light of their already known anti-inflammatory activity and as candidate dual action therapeutics to treat opportunistic fungal infections.

## Introduction

The population of subjects at risk of developing fungal infections is steadily increasing due to rising life expectancy and the continuous medical progress in the treatment of serious diseases such as cancer, transplantation or impairment of immune system (Brown et al., 2012).

Even though advanced medical treatments allow these patients to live longer, the exposure to surgery and medical devices composed of polymeric materials results in evolved ecological niches for biofilm-producing microorganisms and increases the risk for infectious diseases, including those caused by opportunistic fungi (Ramage et al., 2006). *Candida albicans* amongst yeasts and *Aspergillus fumigatus* amongst molds are still the most common pathogens in the clinical setting (Morace and Borghi, 2010; Kriengkauykiat et al., 2011; Guinea, 2014), and continue to carry a high mortality despite the antifungal treatment.

Antifungal resistance is emerging in *Candida* and *Aspergillus* species (Arendrup, 2014), and together with intrinsic or acquired mechanisms, the drug tolerance related to biofilm formation is emerging as having a crucial role in the failure of treatments (Ramage et al., 2014). Fungal cells within the biofilms display resistance to azoles and polyenes, at least at therapeutic doses (Taff et al., 2013). Echinocandins seem to achieve better results against *Candida* biofilms, but not against *A. fumigatus* (Pierce et al., 2013). Thus, the development of new compounds able to overcome the drug-resistance of biofilms is undoubtedly a current and, even more, a future medical need for the treatment of such infections.

Recently, some compounds with known anti-inflammatory properties have been investigated for their antifungal activity. This is of particular relevance in the context of fungal infections. The interplay between fungus and host, i.e., immune system and inflammatory milieu, is crucial in determining the tolerance or the disease status (Romani, 2011). Although inflammation is required to control of fungal infections, its resolution is necessary to avoid collateral damage to tissues and to restore a homeostatic environment (Romani, 2011). Drugs displaying dual activity, antifungal and anti-inflammatory, could thus represent novel approaches to treat biofilm-related infections. In this work we discuss the anti-biofilm properties of myriocin, fulvic acid, and acetylcholine, three compounds recently investigated for their antifungal activity in the context of fungal biofilms.

## Myriocin

Sphingolipids (SPLs) are a class of molecules with structural and signaling activities conserved from fungi to humans. Many studies have demonstrated that SPL mediators are involved in infection-related mechanisms (Mor et al., 2015). Both microbial and mammalian dysregulation of SPLs play a role in the delicate relationship between pathogen and host during the infection process, having an impact on signaling pathways that eventually lead to commensalism or host damage (Heung et al., 2006).

Fungal SPLs have been implicated in several cellular processes such as endocytosis, apoptosis, heat stress response, and fungal pathogenesis (Lattif et al., 2011). In fact, SPLs are part, together with ergosterol, of plasma membrane domains named lipid rafts that are crucial for cell signaling and membrane trafficking, and mediate protein–protein interactions (Farnoud et al., 2015).

Changes in the SPLs content could thus strongly impact the local membrane structure and alter specific protein localization such as the GPI-anchored proteins (Singh and Del Poeta, 2011). These have been extensively studied in *C. albicans* and are crucial for adhesion to substrates in the early phases of biofilm formation (Cabral et al., 2014). Differences in SPLs content have been observed in planktonic and sessile cells of *C. albicans*, suggesting a role for the lipid moiety in biofilm formation and maturation (Lattif et al., 2011). Lipid rafts have been found to localize at the hyphal tip, and drugs affecting SPLs biosynthesis, such as myriocin, lead to defects in hypha formation (Martin and Konopka, 2004).

Myriocin targets the first step of SPLs *de novo* biosynthesis, by inhibiting the enzyme serine palmitoyl transferase (SPT) that catalyzes the condensation of a fatty acyl CoA with serine, a common step to both fungal and mammalian SPLs biosynthesis.

Many cell-stress responses cause ceramide, the central molecule of SPL metabolism, to accumulate and trigger the activation of inflammatory processes (Hannun and Obeid, 2008). High levels of ceramide are characteristic of several inflammatory diseases. Animal models showed that myriocin treatment is able to reduce inflammation by down-regulating ceramide and its related pro-inflammatory cascade (Jiang et al., 2011; Lee et al., 2012; Caretti et al., 2014).

Besides this action and similarly to other SPLs metabolism inhibitors (Groll et al., 1998; Mormeneo et al., 2008), myriocin has a direct antifungal activity (Martin and Konopka, 2004; Lattif et al., 2011; de Melo et al., 2013; Sharma et al., 2014). Recently, Lattif et al. (2011) assessed a potential antibiofilm activity for the drug. The authors grew *C. albicans* biofilms in the presence and absence of various myriocin concentrations and observed a progressive reduction in biofilm biomass and metabolic activity. In addition, lipid raft formation was strongly reduced as well as the *C. albicans* filamentation (Lattif et al., 2011).

Myriocin has been found to be also active against *A. fumigatus* (Cirasola et al., 2014). Administration of myriocin to conidia resulted in a dose-dependent inhibition of germination, whereas the treatment of 24 h pre-formed biofilms strongly reduced the biofilm biomass, as determined by crystal violet assay, and the metabolic activity. In particular, myriocin led to the presence of aberrant hyphal structures in *A. fumigatus*, with increased branching and reduction in apical hyphal growth. Hyphal polarization and branching in *A. fumigatus*, as well as filamentation in *C. albicans*, have been shown to be crucial for virulence and biofilm formation, resulting in more stable biofilms (Brand, 2012; Riquelme, 2013). The inhibition of SPL metabolism disrupts the actin organization at the tip, impacting on normal hyphal growth and differentiation (Cheng et al., 2001). Moreover, a deprived quantity of SPLs results in a decrease of SPLs in lipid rafts with a subsequent reduction of plasma membrane-anchored proteins that participate in the maintenance of polarized growth (Momany, 2002). Although the compound is also active against planktonic fungal cells, all the major SPLs classes seem to be over-represented in the biofilm-organized cells (Lattif et al., 2011), suggesting a key role for SPLs in modulating biofilm formation.

To improve the delivery of myriocin, a highly lipophilic compound, Strettoi et al. (2010) explored the use of solid lipid nanocarriers in a mice model of retinitis pigmentosa. Similarly, other authors observed a decrease in the effective drug concentration compared with pure compound when using nanocarrier delivery in a cystic fibrosis mouse model (Caretti et al., 2014). By treating mice with intratrachea myriocin-loaded nanocarriers, Caretti et al. (2014) were able to achieve a reduction of lung infection and inflammation after *Pseudomonas aeruginosa* infection.

Due to the poor penetration of biofilm matrix by drugs, the same nanocarriers were investigated on fungal biofilms. Nanocarriers improved myriocin delivery into *A. fumigatus* biofilms, allowing its distribution within few hours even in bottom layers (Cirasola et al., 2014).

Due to its dual action, anti-inflammatory and antifungal, myriocin might represent a useful treatment for patients suffering from chronic diseases that increase the risk of fungal infections. However, deeper investigations into its administration need to be performed. Recently de Melo et al. (2013) observed that prophylaxis treatment with myriocin, in an invertebrate model of systemic candidiasis, reduces the insect survival (de Melo et al., 2013). The optimal scenario for the myriocin use could be late phases of fungal infection as well as pathological situations characterized by ceramide mediated hyper-inflammation. On the other hand, the development of myriocin derivatives as well as other compounds targeting downstream steps in the fungal SPL synthesis could increase the specificity of these compounds against fungal enzymes avoiding host side effects.

## Fulvic Acid

Humic substances are commonly found in decaying organic matter including plants, animal residues, sewage and soil (Snyman et al., 2002). Although fulvic acids account for ∼90% of all humic substances and their biological significance recognized for many years (van Rensburg et al., 2000), there is still minimal scientific understanding on which to support the claims of its biological properties. Oxifulvic acid, a derivate of fulvic acid, has been shown to elicit antibacterial and antifungal properties (van Rensburg et al., 2000). However, these formulations contain numerous toxic elements that make their use clinically impossible. Recently, there has been the development of a pure form of fulvic acid, carbohydrate derived fulvic acid (CHD-FA), that has been shown to be safe to use clinically and absent from environmental contaminants known to be harmful to the host (Gandy et al., 2011).

An initial randomized double blind controlled trial indicated that fulvic acid was well-tolerated in patients with eczema, where side effects were minimal and severity and erythema were significantly reduced compared with the placebo control (Gandy et al., 2011). A subsequent phase 1 clinical study carried out to determine the safety profile of CHD-FA, showed that this agent was able to elicit anti-inflammatory properties in addition to being non-toxic when used as an oral formulation (Gandy et al., 2012). This anti-inflammatory activity was also shown in a rat wound model, where the use of a topical cream enhanced wound healing and was non-toxic during both acute and chronic treatments (Sabi et al., 2012). However, so far the mechanism by which CHD-FA elicits the observed immunomodulatory effects is unknown.

Although the anti-inflammatory properties of CHD-FA have been studied, there are very few reports of the antimicrobial properties of this agent. Recent studies have shown CHD-FA to be fungicidal against *C. albicans* planktonic and sessile cells at similar concentrations, indicating good biofilm activity unlike azole antifungals (Sherry et al., 2012). Time-kill analysis of CHD-FA was performed in comparison to the other classes of antifungals, and whilst caspofungin achieved the greatest kill, CHD-FA elicited its maximum activity quicker than any of the other agents, which is of particular benefit in treating systemic infections such as candidemia, where delayed antifungal therapy coincides with mortality rates (Morrell et al., 2005). The rapid killing action was further analyzed by visualizing the uptake of propidium iodide by the cells, only feasible when the cell membrane has been compromised. Membrane damage was recorded as early as 10 min following CHD-FA exposure, which also correlates with the release of intracellular ATP from the cell (Sherry et al., 2012). To further test the hypothesis of a membrane active compound, the activity against the *C. albicans* cell membrane was investigated using a chitin synthase inhibitor. Chitin is a simple polysaccharide found in the cell walls of fungi that provides cell structure and rigidity (Lenardon et al., 2010). It was argued that if the cell membrane was the target of CHD-FA, then by weakening the cell by inhibiting its chitin production would increase the exposure of the cell membrane to the agent and would increase CHD-FA sensitivity (Sherry et al., 2012). Here it was demonstrated that *C. albicans* cells were hyper-susceptible to CHD-FA in the presence of a chitin synthase inhibitor, a finding that was also observed in voriconazole treated biofilms (Kaneko et al., 2010). Collectively, these data suggest that CHD-FA acts through disruption to the cell membrane. It is therefore feasible to suggest that this agent may have broad-spectrum antimicrobial activity against a variety of fungi and bacteria. Indeed, this was the case when CHD-FA was shown to possess antibacterial activity toward a range of oral bacterial biofilms, including an *in vitro* four-species periodontal biofilm model (Sherry et al., 2013).

Additionally, fulvic acid was shown to be minimally affected by characterized biofilm resistance mechanisms, including the extracellular matrix (ECM) and efflux pumps. For example, it is known that glucans within the cellular matrix hinder the penetration of azoles through biofilms, with the depletion of *FKS1*, encoding a β-1,3 glucan synthase, increasing the susceptibility of fluconazole within these communities (Nett et al., 2010a,b). Overexpression of *FKS1*, as well as a deletion mutant, was used to determine the impact of CHD-FA activity. Here it was shown that this agent’s sensitivity was not compromised by the elevated expression of *FKS1*, which is in contrast to azoles, polyenes and echinocandins, where the matrix sequesters these agents and their activity is significantly reduced against *C. albicans* biofilms (Nett et al., 2010a).

Efflux pumps have been widely shown to play a role in azole resistance within *Candida* biofilms, particularly during early biofilm development both *in vitro* and *in vivo* (Ramage et al., 2002; Mukherjee et al., 2003; Nett et al., 2009). Although CHD-FA was shown to induce efflux pump activity in *C. albicans* biofilms, there was no change in the minimum inhibitory concentration (MIC) when an efflux pump inhibitor was used, demonstrating that CHD-FA activity is not compromised by these pumps unlike other antifungals (Sherry et al., 2012).

Overall, whilst our knowledge base for CHD-FA is relatively limited, it does appear to have appropriate biological properties of a broad-spectrum antimicrobial agent and not compromised by know biofilm resistance mechanisms, which has yet undefined immunomodulatory capacity. Further *in vitro* and *in vivo* studies are required to determine its safety profile.

## Acetylcholine

Bi-directional neurochemical interactions occur between the host and colonizing microorganisms (Lyte, 2013, 2014a,b; Sandrini et al., 2015). Many microorganisms share neuro-endocrine mediator synthesis pathways and recognition receptors with their human hosts (Lyte, 2013). Therefore, it is hypothesized that there is constant communication between a vertebrate host and its microbiota, and a bi-directional influence on behavior (Freestone, 2013). However, many of the inter-kingdom signaling molecules and receptors, particularly from the fungal perspective, remain to be characterized in detail. Furthermore, the biological consequences of neuro-endocrine signaling in fungi, with respect to growth and pathogenicity, are only just beginning to be determined.

Acetylcholine (ACh) is widely distributed in both prokaryotic and eukaryotic cells. In mammalian systems, ACh has two major roles: (1) neuronal ACh acts as a neurotransmitter to mediate rapid communication between neurons and effector cells and (2) non-neuronal ACh acts as a local signaling molecule involved in the regulation of cellular phenotype, modification of ciliary activity, and modification of cell-cell contact (Wessler and Kirkpatrick, 2008). In recent years ACh has received greater attention due to the discovery of the “cholinergic anti-inflammatory pathway” that has been demonstrated to regulate immune responses (Borovikova et al., 2000). In this pathway, ACh released from efferent vagus nerve terminals interacts with the alpha 7 nicotinic receptor (α7nAChR) on proximal immune cells resulting in down regulated localized immune responses. In addition, the efferent vagus nerve interacts with the splenic nerve to activate a unique ACh-producing memory phenotype T-cell population, which can propagate ACh mediated immune-regulation throughout the body (Rosas-Ballina et al., 2011). Furthermore, as ACh is also produced by cells out with neural networks, non-neuronal ACh can also play a vital role in localized immune-regulation through its cytotransmitter capabilities (de Jonge et al., 2005; Macpherson et al., 2014). In addition, evidence also suggests that ACh signaling through other cholinergic receptor subtypes, such as the muscarinic receptors, can also modulate inflammatory responses in mammalian systems (Verbout and Jacoby, 2012).

Interestingly, in a recent study, ACh was found to play multiple roles in the pathogenesis of fungal infections in a primitive *Galleria mellonella* infection model. Specifically, ACh was found to: (i) inhibit *C. albicans* yeast–to-hyphae transition and biofilm formation; (ii) promote a rapid and effective cellular immune response to *C. albicans* infection; and (iii) regulate antifungal defenses to limit sepsis induced damage of host tissues (Rajendran et al., 2015). The fact that ACh can directly act on *C. albicans* to inhibit yeast–to-hyphae transition suggests that this organism possesses a functional ACh receptor. However, the ACh receptor(s) and the downstream signaling pathway(s) that are involved in inhibiting *C. albicans* yeast–to-hyphae transition have yet to be characterized in detail.

Sequencing of the *C. albicans* genome has suggested this organism possesses putative cholinergic receptor genes (Inglis et al., 2012). Furthermore, pharmacological evidence suggests that *C. albicans* may possess a receptor that is homologous to human muscarinic (M) receptors. Midkiff et al. (2011) demonstrated that the dopamine receptor antagonist clozapine could inhibit *C. albicans* budding-to-hyphal transition by inhibiting a component of the Efg1 pathway, upstream of the Gpa2 G-alpha subunit, which the authors hypothesized to be the Gpr1 G-protein-coupled receptor (GPCR). However, clozapine has a broad range complex pharmacological profile. Indeed, it is now known that clozapine is a weak dopamine D2 receptor inverse agonist/antagonist and has mixed agonist-antagonist properties on human muscarinic receptors, with strong evidence that it can act as a potent agonist of the M1 and M4 receptors in mammalian systems (Zorn et al., 1994; Olianas et al., 1997, 1999; Miller, 2009; Wiebelhaus et al., 2012). Therefore, it is interesting to speculate that the observed effects on *C. albicans* budded-to-hyphal transition in the study of Midkiff et al. (2011) may be in fact due to clozapine acting upon a putative *C. albicans* cholinergic receptor homologous to human muscarinic receptors. However, further research aimed at characterizing the cholinergic receptor mediated signaling pathways of *C. albicans* is required to confirm this hypothesis.

There is also substantial evidence to suggest that fungi can synthesize and release ACh (Horiuchi et al., 2003; Kawashima and Fujii, 2008). Indeed, sequencing of the *C. albicans* genome revealed this organism to possess putative genes for the enzymes responsible for ACh synthesis; choline acetyltransferase (ChAT) and carnitine acetyltransferase (CrAT; Inglis et al., 2012). However, the ACh synthesis machinery of *C. albicans* remains to be characterized. Furthermore, the biological functions of fungal derived ACh remain to be elucidated.

The fact that both *C. albicans* and its human host both synthesize ACh and possess cholinergic receptors lead to speculate that there is cholinergic mediated bi-directional communication between the two species *in vivo*. The role of this cholinergic bi-directional communication in the maintenance of health and/or the pathogenesis of *C. albicans* infections are at present unknown. The evidence to date suggests the host may utilize ACh to protect against candidiasis (Rajendran et al., 2015). Although, the fact that ACh can modulate host immunity (Tracey, 2010) and also mucosal integrity through the regulation of epithelial cell phenotype and cell–cell contact (Wessler and Kirkpatrick, 2008), may also suggest that *C. albicans* derived ACh may be a potential virulence factor. Either way, further research into the role of bi-directional cholinergic signaling mechanisms between *C. albicans* and the colonized host is required.

The preliminary data to date imply that cholinergic mechanisms may be rational novel therapeutic targets to prevent or treat candidiasis (Rajendran et al., 2015). Indeed, there are a number of pharmacological agonists and antagonists already marketed for the treatment of neurodegenerative disorders, cancers and chronic inflammatory diseases that target cholinergic receptors (Pohanka, 2012; Zoheir et al., 2012; Sales, 2013; Matera and Tata, 2014; Russo et al., 2014). Many of these molecules have already undergone extensive safety and efficacy testing in human trials. Therefore, one or more of these molecules may be worthy of investigation for the prevention or treatment of candidiasis and may offer novel therapeutic approaches beyond conventional antifungals.

## Concluding Remarks

The opportunistic nature of fungal infections highlights the crucial role of the host immune system in regulating host–fungus interactions.

Humans suffer from a range of fungal biofilm diseases that cause high levels of morbidity and mortality. Conventional antifungal drugs have been demonstrated to ineffective against fungal biofilms, and alternative strategies are needed to overcome their intrinsic resistance.

Therefore molecules targeting both fungal biofilm formation and the host inflammatory response could represent a new therapeutic approach to treat fungal biofilm-related infections with broader implications for healthcare applications.

### Conflict of Interest Statement

The authors declare that the research was conducted in the absence of any commercial or financial relationships that could be construed as a potential conflict of interest.
